# Isotianil (2^nd^ edition) (Pesticides)

**DOI:** 10.14252/foodsafetyfscj.D-23-00014

**Published:** 2023-12-22

**Authors:** Food Safety Commission of Japan

## Abstract

Food Safety Commission of Japan (FSCJ) conducted a risk assessment of isotianil (CAS No. 224049-04-1), an isothiazole agent for induced resistance to blast disease. This evaluation was requested from Ministry of Agriculture, Forestry and Fisheries (MAFF) on the reevaluation article of Agricultural Chemicals Regulation Act. Additional information including the fate in livestock (goats and chickens) and genotoxicity, and also the list of published scientific literature were newly submitted from the MAFF. The following data were used in the assessment; fate in plants (including paddy rice and potatoes), residues in crops, fate in livestock (goats and chickens), residues in livestock products, fate in animals (rats), subacute toxicity (rats, mice and dogs), chronic toxicity (rats and dogs), carcinogenicity (rats and mice), two-generation reproductive toxicity (rats), developmental toxicity (rats and rabbits), and genotoxicity. Major adverse effects of isotianil were observed in the stomach (mucosal epithelium hyperplasia of the forestomach limiting ridge in rats), the liver (including organ weight gain), and the kidney (including chronic nephropathy). No adverse effects were observed on carcinogenicity, fertility, teratogenicity, and genotoxicity. The lowest no-observed-adverse-effect level (NOAEL) obtained from the studies described above was 2.83 mg/kg bw per day in a one-year chronic toxicity study in rats. FSCJ specified an acceptable daily intake (ADI) of 0.028 mg/kg bw per day based on the NOAEL after applying a safety factor of 100. An acute reference dose (ARfD) was judged unnecessary to be specified, based on the results of a single oral administration of isotianil and other related tests.

## Conclusion in Brief

Food Safety Commission of Japan (FSCJ) conducted a risk assessment of isotianil (CAS No. 224049-04-1), an isothiazole agent for induced resistance to blast disease. This evaluation was requested from Ministry of Agriculture, Forestry and Fisheries (MAFF) on the reevaluation article of Agricultural Chemicals Regulation Act. Additional information including the fate in livestock (goats and chickens) and genotoxicity, and also the list of published scientific literature were newly submitted from the MAFF.

The following data were used in the assessment; fate in plants (including paddy rice and potatoes), residues in crops, fate in livestock (goats and chickens), residues in livestock products, fate in animals (rats), subacute toxicity (rats, mice and dogs), chronic toxicity (rats and dogs), carcinogenicity (rats and mice), two-generation reproductive toxicity (rats), developmental toxicity (rats and rabbits), and genotoxicity.

Major adverse effects of isotianil were observed in the stomach (mucosal epithelium hyperplasia of the forestomach limiting ridge in rats), the liver (including organ weight gain), and the kidney (including chronic nephropathy). No adverse effects were observed on carcinogenicity, fertility, teratogenicity, and genotoxicity.

Based on these results, parent isotianil only was identified as the relevant substance for the residue definition for dietary risk assessment in agricultural products, livestock products and fishery products.

The lowest no-observed-adverse-effect level (NOAEL) obtained from the studies described above was 2.83 mg/kg bw per day in a one-year chronic toxicity study in rats. FSCJ specified an acceptable daily intake (ADI) of 0.028 mg/kg bw per day based on the NOAEL after applying a safety factor of 100.

An acute reference dose (ARfD) was judged unnecessary to be specified, based on the results of a single oral administration of isotianil and other related tests.(table 1)

**Table 1.** *Levels relevant to toxicological evaluation of isotianil*

Species	Study	Dose (mg/kg bw per day)	NOAEL (mg/kg bw per day)	LOAEL (mg/kg bw per day)	Critical endpoints^1^^)^
Rat	90-day subacute toxicity study	0, 20, 500, 2 500, 20 000 ppm	M: 29.7 F: 35.1	M: 148 F: 178	M/F: Increased T.Chol levels, etc.
		M: 0, 1.18, 29.7, 148, 1 240 F: 0, 1.39, 35.1, 178, 1 400			
	One-year chronic toxicity study	0, 60, 600, 6 000, 20 000 ppm	M: 2.83 F: 3.70	M: 27.9 F: 37.3	M/F: Increased T.Chol levels and increased relative weight of the liver
		M: 0, 2.83, 27.9 291, 979 F: 0, 3.70, 37.3, 381, 1 250			
	Two-year carcinogenicity study	0, 2 000, 6 000, 20 000 ppm	M: - F: -	M: 79.2 F: 105	M/F: Mucosal epithelium hyperplasia of the forestomach limiting ridge F: Chronic nephropathy (No carcinogenicity is observed)
		M: 0, 79.2, 242, 823 F: 0, 105, 311, 1 050			
	Two-generation reproductive toxicity study	0, 50, 1 000, 10 000 ppm	Parent PM: 3.35 PF: 4.16 F_1_M: 4.05 F_1_F: 4.74 Offspring PM: 3.35 PF: 4.16 F_1_M: 4.05 F_1_F: 4.74	Parent PM: 66.8 PF: 83.9 F_1_M: 80.6 F_1_F: 95 Offspring PM: 66.8 PF: 83.9 F_1_M: 80.6 F_1_F: 95	Parent M/F: Increased absolute and relative weights of the liver, etc. Offspring: Low body weight (No effect on fertility is observed)
		PM: 0, 3.35, 66.8, 662 PF: 0, 4.16, 83.9, 831 F_1_M: 0, 4.05, 80.6, 823 F_1_F: 0, 4.74, 95, 941			
	Developmental toxicity study	0, 100, 300, 1 000	Dams: 1 000 Fetuses: 1 000	Dams: - Fetuses: -	Dams and fetuses: No toxicity (No teratogenicity observed)
Mouse	90-day subacute toxicity study	0, 150, 1 000, 7 000 ppm	M: 1 310 F: 2 470	M: - F: -	M/F: No toxicity
		M: 0, 33.1, 204, 1 310 F: 0, 54.8, 401, 2 470			
	18-month carcinogenicity study	0, 70, 700, 7 000 ppm	M: 706 F: 667	M/F: -	M/F: No toxicity (No carcinogenicity is observed)
		M: 0, 6.89, 71.5, 706 F: 0, 6.66, 67.2, 667			
Rabbit	Developmental toxicity study	0, 100, 300, 1 000	Dams: 300 Fetuses: 300	Dams: 1 000 Fetuses: 1 000	Dams: Suppressed body weight gain, etc. Fetuses: Low body weight (No teratogenicity observed)
Dog	90-day subacute toxicity study	0, 500, 2 000, 8 000 ppm	M: 12.2 F: 13.4	M: 51.1 F: 54.4	M/F: Increased ALT, etc.
		M: 0, 12.2, 51.1, 200 F: 0, 13.4, 54.4, 211			
	One-year chronic toxicity study	0, 200, 1 000, 5 000/3 000 ppm	M: 5.22 F: 5.33	M: 27.2 F: 26.9	M: Increased in absolute and relative weights of the liver, etc. F: Increase in absolute weight of the spleen, etc.
		M: 0, 5.22, 27.2, 107 F: 0, 5.33, 26.9, 110			
ADI			NOAEL: 2.83 SF: 100 ADI: 0.028		
The critical study for setting ADI			One-year chronic toxicity study (rat)		

ADI, Acceptable daily intake; ALT, Alanine aminotransferase; NOAEL, No-observed-adverse-effect level; SF, Safety factor; T. Chol, Total cholesterol; -, LOAEL could not be specified.

^1)^ The adverse effect observed at LOAEL
